# Dysregulated Alternative Splicing Pattern of PKC**δ** during Differentiation of Human Preadipocytes Represents Distinct Differences between Lean and Obese Adipocytes

**DOI:** 10.1155/2013/161345

**Published:** 2013-04-10

**Authors:** Gay Carter, André Apostolatos, Rekha Patel, Abhishek Mathur, Denise Cooper, Michel Murr, Niketa A. Patel

**Affiliations:** ^1^James A. Haley Veterans' Hospital, Research Service VAR 151, 13000 Bruce B. Downs Boulevard, Tampa, FL 33612, USA; ^2^Department of Molecular Medicine, Morsani College of Medicine, University of South Florida, Tampa, FL 33612, USA; ^3^Department of Surgery, Morsani College of Medicine, University of South Florida, Tampa, FL 33612, USA

## Abstract

Obesity and its comorbidities affect millions of people. Here, we demonstrate that human preadipocytes are susceptible to programmed cell death (apoptosis) while mature adipocytes are resistant to apoptosis. The molecular mechanisms underlying the phenotype of apoptosis-resistant adipocytes are lesser known. To study the role of apoptosis and define molecular differences in the developmental process of adipogenesis, human preadipocytes were differentiated *in vitro* to mature adipocytes. Many genes in the apoptosis pathway are alternatively spliced. Our data demonstrates that during differentiation PKC**δ**, Bclx, and Caspase9 switch to their prosurvival splice variants along with an increase in Bcl2 expression when the cells terminally differentiate into mature adipocytes. Next we determined the expression pattern of these genes in obesity. Our data indicated high expression of PKC**δ**VIII in adipose tissue of obese patient in different depots. We demonstrate a shift in the *in vitro* expression of these splice variants in differentiating preadipocytes derived from obese patients along with a decrease in adipogenesis markers. Hence, the programmed splicing of antiapoptotic proteins is a pivotal switch in differentiation that commits adipocytes to a prosurvival pathway. The expression pattern of these genes is dysregulated in obesity and may contribute to adipose tissue dysfunction.

## 1. Introduction

The human proteome is changing constantly in response to hormones, age, and developmental stage or disease. The genetic makeup of the body indicates about 25,000 genes responsible for close to 100,000 proteins in a given proteome. Alternative splicing is a quintessential mechanism to generate proteins with distinct functions from the same gene. Alternative splicing occurs in more than 90% of genes and is a powerful step in gene expression to diversify the genomic repertoire. 

Genetic, environmental, and cultural factors contribute to the onset of obesity. In order to develop a therapeutic agent to combat obesity, it is essential to understand the molecular mechanisms underlying adipogenesis. Differentiation of preadipocytes to mature adipocytes is usually studied in 3T3L1 and 3T3F442A murine preadipocyte cell lines as they reproduce adipogenesis *in vitro* including expression of adipogenic genes and morphological changes. However, beyond the obvious species differences, preadipocytes from mouse and humans show differences as shown by gene-centric analysis of adipogenesis marker genes such as PPAR*γ* and C/EBP*α*, **β** and **δ** [1]. It is also known that, unlike murine adipocytes, human preadipocytes do not require clonal expansion to differentiate into adipocytes *in vitro. *Hence, we sought to elucidate the gene expression patterns during human adipogenesis which dictate the mature adipocyte phenotype. Previous studies on adipogenesis have focused on transcriptional control in adipocyte differentiation. Additional mechanisms of gene expression which are required to maintain the adipocyte phenotype are unknown. 

Cell death is achieved by several mechanisms including necrosis (sudden cell death, fatally affecting neighboring cells), apoptosis (programmed cell death), and autophagy (large scale degradation via lysosomes). It is now established that loss of adipose tissue mass in rodents and humans is achieved by inducing apoptosis [2–6]. Caspases directly and indirectly orchestrate the morphologic changes of the cell during apoptosis. Caspases have been characterized into upstream initiators (caspase 2, 8, 9, 10) and downstream executioners (caspase 3, 6, 7) [7]. Bcl-2 protein family has about 17 members and the decision of a cell to undergo apoptosis is governed by a complex network of interactions between these Bcl-2 members, caspases, Smac/DIABLO (second mitochondria-derived activator of caspase), and X-linked inhibitor of apoptosis (XIAP). The prosurvival subfamily comprises of Bcl-2, Bcl-xL, Bcl-w, Mcl-1, A1, and human Bcl-B. The release of Smac/DIABLO from the mitochondria is regulated by antiapoptotic Bcl-2 proteins—Bcl-2 and Bcl-xL. Hence, the decision to undergo apoptosis is a cascade of events and proteins that work in concert. PKC*δ* is a signaling kinase affecting downstream apoptotic pathways. Our laboratory has demonstrated that the alternatively spliced products of human PKC*δ* have distinct functions in apoptosis. PKC*δ*I promotes apoptosis while PKC*δ*VIII is antiapoptotic [8].

Earlier studies in mouse models indicated that preadipocytes can undergo programmed cell death (apoptosis). Here, we demonstrate that human preadipocytes are susceptible to apoptosis while mature adipocytes are resistant to apoptosis. Many genes in the apoptosis pathway are alternatively spliced. We sought to determine the alternative splicing patterns of genes in the apoptosis pathway which promote the antiapoptotic nature of the mature adipocytes. This contributes to the inability of an individual to maintain weight loss as diet and exercise may reduce the size of the fat cells but the cells do not die and can easily gain back the fat mass. The excess energy (input > expenditure) is stored in the white adipose tissue (WAT) which is also an important endocrine regulator.

## 2. Materials and Methods

### 2.1. Human Preadipocytes

The cells were purchased as cryopreserved preadipocytes from ZenBio (Research Triangle Park, NC, USA). Since these commercial cell lines and their information are publicly available, they are exempt from human subjects research determination from IRB in accordance with HHS regulation at 45 CFR Part 46. The lean preadipocytes originated from subcutaneous adipose tissue of a healthy donor of 26 years of age undergoing elective surgery with a body mass index of 21.3 who was not diabetic and not a smoker. The obese preadipocytes originated from subcutaneous adipose tissue of a 40-year-old donor undergoing elective surgery with a body mass index of 54.6 who was not diabetic and not a smoker. The preadipocyte cell line (prescreen by ZenBio) is sorted and characterized such that it represents only preadipocytes and is free from stem cells or differentiating adipocytes or contaminating endothelial cells. Both the preadipocytes are tested in culture to differentiate into mature adipocytes and show accumulation of lipid, express aP2, respond to lipolytic agents and secrete adiponectin and leptin. At the start of all experiments, cells are grown to confluency such that all cells are synchronized and then differentiated. The cells were cultured according to the manufacturer's instructions. Briefly, cryopreserved preadipocytes were passaged with preadipocyte medium (PM-1; DMEM/Ham's F-12 medium, HEPES, FBS, penicillin, streptomycin, amphotericin B; Zen-Bio) and then plated 40,625 cells/cm^2^ with PM-1. Cells were fed every other day with PM-1 until confluent. To induce differentiation, PM-1 medium was replaced with differentiation medium (DM2; Zen-Bio) including biotin, pantothenate, human insulin, dexamethasone, isobutylmethylxanthine, and a PPAR*γ* agonist (days 0–7). After 7 days, DM-2 medium was removed and cells were incubated for additional 7 days with adipocyte medium (AM1; Zen-Bio; days 7–14), which included PM-1, biotin, pantothenate, human insulin, and dexamethasone. By day 14, cells contained large lipid droplets and were considered mature adipocytes. Cells were maintained at 37°C in a humidified 5% CO_2_ atmosphere.

### 2.2. Adipose Lysates

We obtained protein lysates from Drs. Murr and Cooper, USF and VAMC, Tampa. The protein lysates were harvested from adipose tissues in Dr. Cooper's lab and originated from subcutaneous and omental adipose depots from an obese patient. The tissues were obtained under consent from a deidentified patient, age 45, nondiabetic, BMI 56 undergoing Roux-en-y gastroenterostomy surgery. The fat collection protocols were approved by the Institutional Review Board of the University of South Florida Ethics Committee protocol number 108360.

### 2.3. Quantitative Real-Time RT-PCR

Total RNA was isolated from differentiated lean and obese preadipocytes using RNAbee according to the manufacturer's protocol (TelTest Inc.). 2 *μ*g RNA was reverse transcribed using Qiagen's RT kit. 2 *μ*L of cDNA was amplified by real-time quantitative PCR using Syber (SYBR) Green with an ABI PRISM 7900 sequence detection system (PE Applied Biosystems) to quantify the absolute levels of the transcripts in the samples. *β*-actin was used as the endogenous control. Two sets of primer pairs for each transcript FOXO1, SIRT1, PPAR*γ*, and adiponectin were designed to have an annealing temperature of ~60°C. Primers for PKC*δ*VIII are described in our earlier publication [9]. These primers were initially tested using cDNA from human preadipocytes in an RT-PCR reaction using Taq polymerase to give distinct products corresponding to the respective transcripts. Next, the optimal primer concentration was determined from a range of 50–900 nM. The final concentration of 300 nM was selected to ensure efficiency and specificity for its target based on the dissociation curve that showed a single, sharp peak, indicating that the primers amplify one specific target. For absolute quantification, a standard curve was generated for each gene in every assay. To do so, 100–0.4 ng of RNA was reverse transcribed as described above. The resulting cDNA was used to obtain a standard curve correlating the amounts with the threshold cycle number (Ct values). A linear relationship (*r* ^2^ > 0.96) was obtained for each gene. Real-time PCR was then performed on samples and standards in triplicate. The plate setup also included a standard series, no template control, no RNA control, no reverse transcriptase control, and no amplification control. The dissociation curve was analyzed for each sample. Absolute quantification of mRNA expression levels for individual transcripts was calculated by normalizing the values to GAPDH. The results were analyzed with a two-tailed Student's *t*-test using PRISM4 statistical analysis software (GraphPad). A level of *P* < 0.05 was considered statistically significant. Significance was determined after three or more experiments.

### 2.4. Western Blot Analysis

Protein lysates were obtained from the cells using lysis buffer containing protease inhibitors. Protein lysates were also harvested from the snap-frozen adipose tissues by homogenization and sonication in the lysis buffer. The lysates (40 *μ*g) were separated by SDS-PAGE with 10% gels, electrophoretically transferred to nitrocellulose membranes, blocked with Tris-buffered saline containing 0.1% Tween 20 and 5% nonfat dried milk, washed, and incubated with anti-PKC*δ* or antibody specific for PKC*δ*VIII (Patel lab [8]), anti-PPAR*γ*, anti-adiponectin, anti-Bcl2, anti-caspase 9 (Cell Signaling), anti-XIAP (AnaSpec), anti-Bcl-xL (Santa Cruz Biotechnology), and *β*-actin (Sigma). After incubation with antirabbit IgG-HRP, enhanced chemiluminescence (Pierce) was used for detection. 

### 2.5. Adipocyte Size

Adipocytes were differentiated in 100 mm plate and their sizes determined on day 14 (i.e., of mature adipocytes). Maximal diameter of 10 adjacent adipocytes from 6 different fields was calculated using Nikon Eclipse inverted microscope and NIS elements advance research image tool software. The data was transferred to Excel to calculate the mean diameter and standard deviation. This was repeated in three separate experiments to ensure reproducibility.

### 2.6. Apoptosis Assay

Human preadipocytes were cultured on 60 mm dishes as described in methods. For apoptosis assays, cells were serum starved for 48 hours. Media were collected and cells were washed one time with HBSS and then trypsinized for 5.0 minutes. Five ml complete media was added to neutralize the trypsin. Media and washes were pooled and centrifuged at 1200 RPMS for five minutes. Cells were washed one time with PBS and one time with binding buffer and then incubated for 15 min with 5.0 *μ*L AV-FITC and 5.0 *μ*L PI in 100 uL binding buffer (BD Pharmagen, San Diego, CA, USA) at room temperature in the dark. 400 *μ*L binding buffer was added and cells were analyzed by flow cytometry within one hour. Annexin V-FITC and PI fluorescence were measured using an Accuri C6 flow cytometer. 

### 2.7. Statistical Analysis

Analyses were performed using PRISM software and analyzed using either two-tailed Student's *t*-test or two-way ANOVA. *P < 0.05 was significant; ***P < 0.0001 was highly significant. Analysis was performed within group and between groups.

## 3. Results

### 3.1. Human Preadipocytes Are Susceptible to Apoptosis

**Figure 1:**
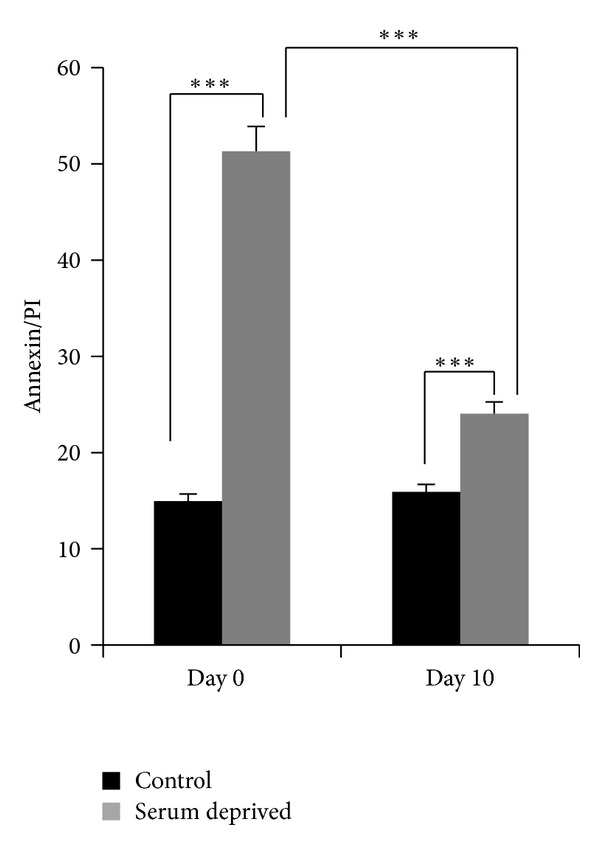
Human lean preadipocytes were differentiated *in vitro*. Cells were serum deprived for 48 h on day 0 (preadipocytes) and on day 10 (mature adipocytes). Annexin/PI was analyzed by flow cytometry. Experiments were repeated 5 times. Statistical analysis performed by two-way ANOVA; *P* > 0.75 ns, not significant within group; ***P < 0.0001 highly significant between control and serum-deprived; ***P < 0.0001 highly significant between days 0 and 10 serum-deprived.

Human preadipocytes were commercially obtained from ZenBio such that a pure population of preadipocytes devoid of contaminating stem cells or adipocytes or other nonadipocyte lineage cells were used in our experiments. The cells were cultured as described in methods. Cells were serum starved for 48 hours to induce apoptosis and assayed by flow cytometry on days 0 and 10. FITC Annexin V was used to quantitatively assess cells undergoing apoptosis along with propidium iodide to enable detection of percentage of cells undergoing either early apoptosis or late apoptosis. Annexin V binds to phosphatidylserine which is displayed on the cell membrane of apoptotic cells and PI will stain only dead or damaged cells. The results (Figure 1) demonstrate that on day 0 preadipocytes which are serum starved undergo increased apoptosis while the mature adipocytes (day 10) are more resistant to apoptosis upon serum starvation. 

### 3.2. Differentiation of Human Preadipocytes Demonstrates Switch in Apoptosis Genes

Since the adipocytes demonstrated a switch in the susceptibility to apoptosis during differentiation, we sought to elucidate the expression patterns of apoptotic genes during differentiation. We evaluated the alternative splicing of genes during differentiation with a focus on apoptotic genes. We used gene-level exon array from Affymetrix (experiments performed by Microarray Core Facility, Moffitt Cancer Institute, and analyzed with Exon array analyzer software to sort specific biochemical pathways and genes) to identify a group of transcripts that are predominantly alternatively spliced and expressed. We used days 0, 6, and 8 of differentiating human preadipocytes (data not shown). The splicing pattern of genes of the apoptosis pathway that showed a dramatic change between days 0 and 6 of adipogenesis is explained below. 

### 3.3. Alternative Splicing of PKC*δ*

**Figure 2:**
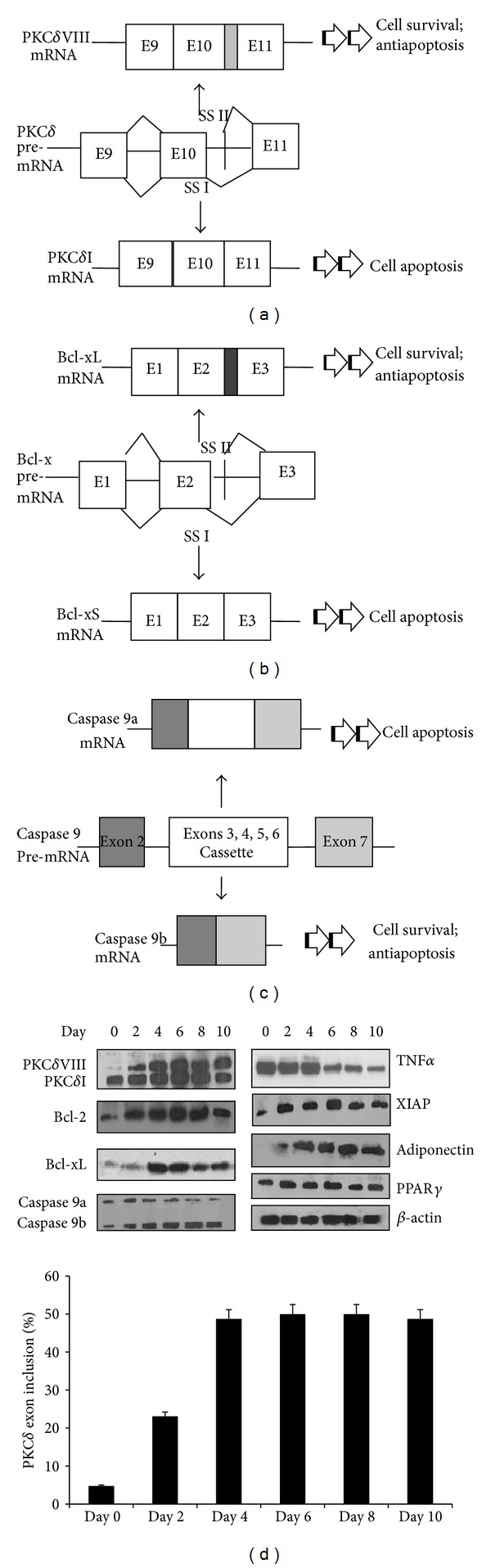
(a) Schematic of alternative splicing of PKC*δ* pre-mRNA generating PKC*δ*I mRNA and PKC*δ*VIII mRNA via alternative 5′ splice site selection. E: exon; SS: splice site. (b) Schematic of alternative splicing of Bcl-x pre-mRNA generating Bcl-xL mRNA and Bcl-xS mRNA via alternative 5′ splice site selection. E: exon; SS: splice site. (c) Schematic of alternative splicing of caspase 9 pre-mRNA generating caspase 9a mRNA and caspase 9b mRNA via cassette exon inclusion. (d) Western blot analysis of differentiating lean preadipocytes from days 0 to 10 using antibodies as indicated in the figure. Between days 2 and 4, a marked shift is observed in the splicing pattern of survival proteins. This period marks terminal differentiation of adipocytes. The blots are representative of 3 experiments performed individually with similar results. Graph represents percent PKC*δ* exon inclusion calculated as PKC*δ*VIII/(PKC*δ*VIII + PKC*δ*I) × 100 and is representative of four experiments performed separately.

Protein kinase C delta (PKC*δ*) is a serine/threonine kinase which plays a central role in apoptosis. Previously, we identified a new splice variant of human PKC*δ*, PKC*δ*VIII (Figure 2(a)). Sequencing and computational analysis of the PKC*δ*VIII sequence indicated that this human splice variant is generated by utilization of an alternative downstream 5′ splice site of PKC*δ* pre-mRNA exon 10. Our data indicated that PKC*δ*I promotes apoptosis while PKC*δ*VIII promotes cell survival [8].

### 3.4. Alternative Splicing of Bcl-x

Bcl-x is a dominant regulator of programmed cell death in mammalian cells. Bcl-x is alternatively spliced to Bcl-xL, Bcl-xS, and Bcl-x*β*. Bcl-xL is generated via alternate 5′ splice site selection on exon 2 of Bcl-x pre-mRNA and includes the BH1 and BH2 domains (Figure 2(b)). Bcl-x*β* is the least studied and has no known role in apoptosis. The long form Bcl-xL inhibits apoptosis while the short isoform Bcl-xS promotes apoptosis. Bcl2 and Bcl-xL dimerize to initiate the cell survival pathways. 

### 3.5. Alternative Splicing of Caspase 9

Caspase 9, an initiator caspase, is a ubiquitously expressed protease. It represents a pivotal signaling protein in the apoptotic cascade. Inhibition of Caspase 9 by a chemical inhibitor mollugin affects adipogenesis [10, 11]. It is alternatively spliced to caspase 9a (apoptotic) and caspase 9b (antiapoptosis) via inclusion of the cassette exons 3, 4, 5, and 6 in caspase 9a (Figure 2(c)). Caspase 9a activates caspase 3 which cleaves substrates thereby mediating apoptosis. 

To verify our results from the exon array, we carried out a detailed analysis of the differentiation of human preadipocytes from days 0 to 10 as they differentiate into mature adipocytes as described. Our data indicates that between days 2 and 4 of differentiation a marked shift is observed in the expression of genes involved in prosurvival pathways (Figure 2(d)). Day 6 of differentiation is marked by the splicing of antiapoptotic proteins that advances the mature adipocytes to be resistant to apoptosis. On day 0, cells expressed the apoptotic splice variants PKC*δ*I, caspase 9a, and lower levels of Bcl-xL. By day 4, cells started expressing the antiapoptotic proteins Bcl2 and splice variants PKC*δ*VIII, caspase 9b, and Bcl-xL with a concurrent decrease in proapoptotic proteins. This represents a critical switch in adipocyte differentiation modulated by the splicing of apoptosis genes. 

### 3.6. Expression of PKC*δ* Splice Variants in Obese Patients

**Figure 3:**
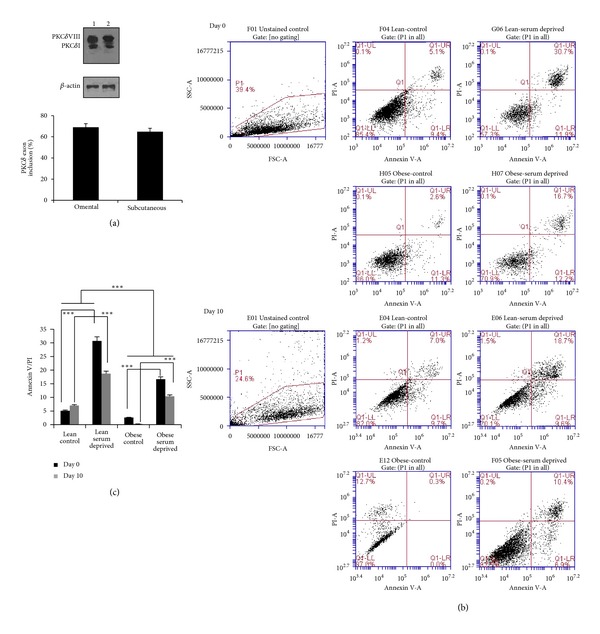
(a) Western blot analysis of adipose tissue lysates obtained from obese depots from (1) omental; (2) subcutaneous. The blots are representative of experiments repeated thrice from the lysates. Graph represents percent PKC*δ* exon inclusion calculated as PKC*δ*VIII/(PKC*δ*VIII + PKC*δ*I) × 100 and is representative of three experiments. (b) Human lean and obese preadipocytes were differentiated *in vitro*. Cells were serum deprived for 48 h on day 0 (preadipocytes) and on day 10 (mature adipocytes). Annexin V/PI staining was analyzed by flow cytometry. The first plot demonstrates the gating of the adipocyte population. The remaining plots shown are lean control, lean-serum deprived, obese control, and obese serum-deprived and are divided into four quadrants based on Annexin V (AV) and propidium iodide staining (PI) staining. Q1-LL represents viable (unstained) cells. Q1-LR represents AV staining only (early apoptosis). Q1-UR represents cells stained with both AV and PI (later apoptosis). Q1-UL represents cells stained with PI only (end-stage apoptosis). Data shown are representative of five different experiments. (c) Graph represents AV/PI and represents 5 experiments. Statistical analysis performed by two-way ANOVA; *P* > 0.75 ns, not significant within group; ***P < 0.0001 highly significant between control and serum-deprived; ***P < 0.0001 highly significant between lean and obese; ***P < 0.0001 highly significant between days 0 and 10 serum-deprived.

Our above data was obtained in normal, lean differentiating preadipocytes. Next, we sought to determine whether the splicing pattern and expression of these genes was affected by the metabolic state. Hence, we sought to measure the levels of PKC*δ* from obese patients. We obtained protein lysates from subcutaneous and omental adipose tissues from an obese patient undergoing Roux-en-y gastroenterostomy surgery (from Drs. Murr and Cooper, Tampa VAMC). The samples were from an obese patient closely matching the BMI and other criteria to the preadipocytes obtained from ZenBio to allow for a better comparison. The results indicated a sharp increase in PKC*δ*VIII expression in the obese patient compared to PKC*δ*I (Figure 3(a)). It is important to note that these adipose tissue samples represent a heterogeneous cell population containing predominantly mature adipocytes and also differentiating preadipocytes as well as other stem cells which may or may not have the adipocyte lineage. 

Next, we obtained preadipocytes from the subcutaneous depot of obese patients (ZenBio) and differentiated them *in vitro*. We chose to further study the preadipocyte differentiation of obese subcutaneous fraction because our lean cell line was derived from the subcutaneous fraction. The cell line was selected to match the adipose tissue lysate (above) such that we could evaluate differentiation of obese preadipocytes *in vitro*. It also allowed us to compare the expression patterns of the genes between the lean and obese during differentiation. We first evaluated apoptosis and susceptibility to serum deprivation on days 0 and 10 by annexin V and PI flow cytometry. Our results (Figures 3(b) and 3(c)) indicated that obese preadipocytes are more resistant to apoptosis compared to lean on day 0 (UR quadrant with both AV and PI: lean 30.7%; obese 16.7%). Day 10 mature adipocytes demonstrate much lower susceptibility to serum-deprived apoptosis (lean 18.7%; obese 10.4%). 

**Figure 4:**
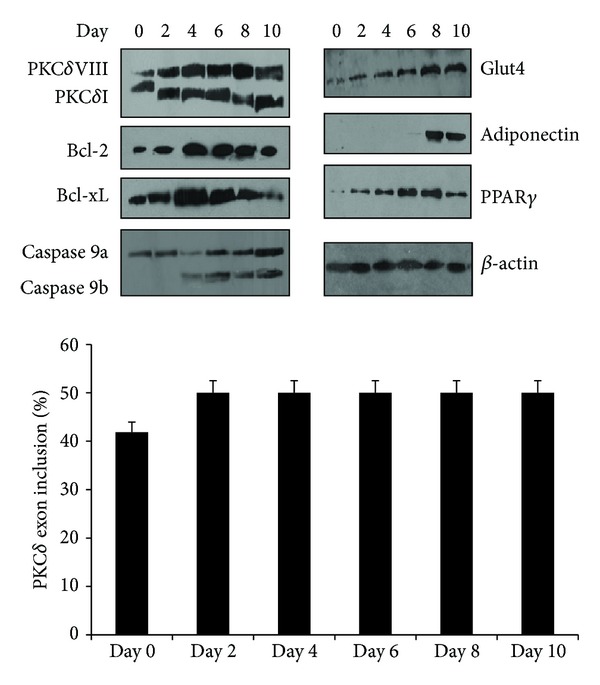
Western blot analysis of differentiating subcutaneous obese preadipocytes from days 0 to 10 using antibodies as indicated. The blots are representative of four experiments performed individually with similar results. Graph represents percent PKC*δ* exon inclusion calculated as PKC*δ*VIII/(PKC*δ*VIII + PKC*δ*I) × 100 and is representative of four experiments performed separately.

To determine the splicing pattern of differentiating obese preadipocytes, we performed Western blot analysis. We observed that these cells expressed PKC*δ*VIII, Bcl-xL, Bcl2, and caspase 9 on day 0, that is, in the preadipocyte stage. We observed increased expression of PKC*δ*VIII as determined by the PKC*δ* splice variant ratio observed on day 0 (Figure 4). This data suggests that the early expression of these genes in the adipocytes promotes their survival mechanisms and thereby provides resistance to cell death. 

### 3.7. Adipogenesis in Obesity Is Dysregulated

**Figure 5:**
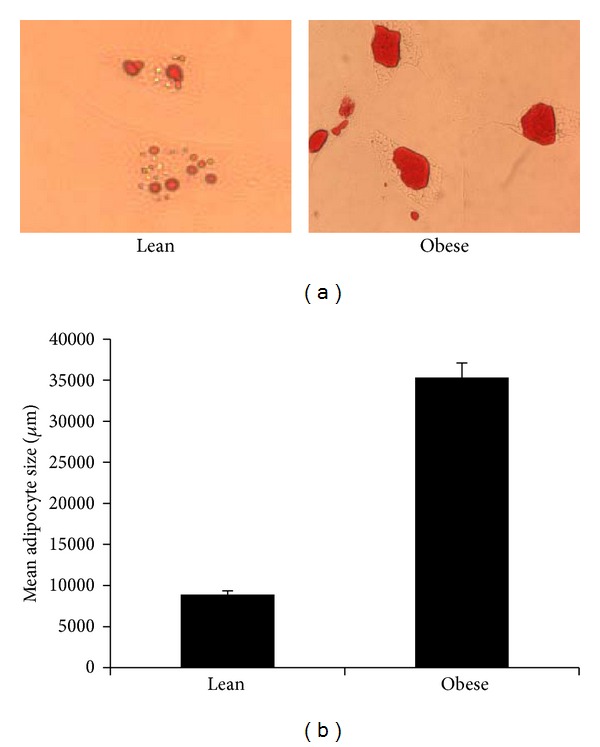
Human lean or obese preadipocytes were differentiated *in vitro*. (a) Adipocytes on day 14 were stained for lipid content with Oil Red O and (b) adipocyte size measured (methods). Experiments were repeated 4 times with similar results.

The lean and obese human preadipocytes were differentiated for 0 to 14 days and adipogenesis was observed by staining of the lipid droplets by Oil Red O. The lean cells showed lipid accumulation beginning at day 4. We observed that obese preadipocytes were slower to differentiate but exhibited larger lipid droplets by day 10. We measured and compared the mature adipocyte size on day 14 between lean and obese samples (Figure 5). Our results indicate a dramatic increase in adipocyte size in obese samples. Further, the expression of adiponectin and PPAR*γ* was dysregulated in differentiating obese preadipocytes (Figure 4) which may explain the aberrant differentiation and adipogenesis observed in obese patients. These results indicated that the adipogenesis program was dysregulated in obese adipocytes. 

**Figure 6:**
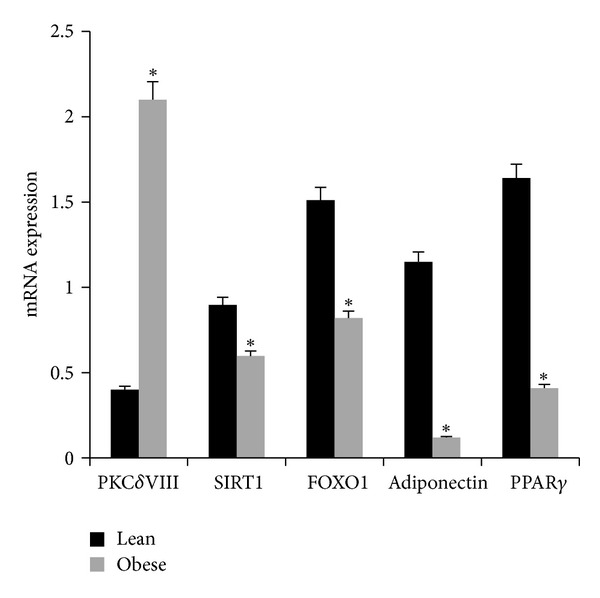
Lean and obese human preadipocytes were differentiated *in vitro*. Total RNA was collected on day 2. Real Time SYBR qPCR analysis of PKC*δ*VIII, SIRT1, FOXO1, adiponectin, and PPAR*γ* was performed in triplicate and repeated 3 times in separate experiments. The absolute mRNA expression transcripts are shown. *P < 0.05 (by two-tailed Student's *t*-test) significant between lean and obese for each mRNA.

To evaluate this, we performed real-time qPCR analysis to measure the expression of adipogenesis marker genes FOXO, SIRT1, adiponectin, and PPAR*γ* with a concurrent measure of PKC*δ*VIII expression. Total RNA was isolated on day 2 of differentiation of lean and obese preadipocytes. Our results (Figure 6) indicated that PKC*δ*VIII expression was increased dramatically in obese samples along with a marked decrease in FOXO, SIRT1, adiponectin, and PPAR*γ* expression compared to lean samples.

## 4. Discussion

Obesity is an epidemic facing the world with the USA leading the statistics on increasing number of overweight and obese population. Adipose tissue is an important endocrine regulator of energy homeostasis and glucose metabolism and the excess energy is stored in the white adipose tissue. The onset of adipogenesis and the transcriptional factors that initiate it have been studied in depth. However, with our current knowledge of the genome, it is evident that there are several other mechanisms in play which regulate the adipocyte phenotype during differentiation. Apoptosis is required for fat cell turnover throughout our life. However, for weight loss in obese patients it may be beneficial to increase adipocyte apoptosis along with therapies targeting hyperplasia and hypertrophy in adipocytes. To our knowledge, this is the first demonstration of alternative splicing events during adipogenesis which contribute to the mature adipocyte phenotype. Our results indicate a distinct splicing pattern between lean and obese patients. 

Apoptosis in preadipocytes and adipocytes was previously observed in human cell lines. However, the underlying mechanisms were not described. Alternative splicing, a posttranscriptional event, is a powerful regulator of gene expression. We have identified key genes which are alternatively spliced that dramatically switch their splice variant expression during the progression of adipogenesis. This may explain why preadipocytes are more susceptible to apoptotic agents while the mature adipocytes are resistant to it. Protein kinase C delta (PKC*δ*) is a crucial signaling kinase affecting downstream apoptotic pathways [8, 12]. Our data indicates increased expression of PKC*δ*VIII along with an early switch between the splice variants associated with apoptosis during adipogenesis process of preadipocytes derived from obese patients.

PKC*δ* is a serine/threonine kinase which plays a central role in apoptosis. PKC*δ* has dual functions: as a mediator of apoptosis and as an antiapoptosis effector. Its splice variants function as a switch that determines cell survival and fate. This could explain the opposing effects of PKC*δ* on cellular apoptosis cited in the literature [13–15]. Genetic switches based on alternative splicing are important for many cellular and developmental processes. PKC*δ*I promotes apoptosis while PKC*δ*VIII promotes survival. We have shown that PKC*δ*II (the mouse splice variant) and PKC*δ*VIII function as prosurvival proteins [8, 12]; the functions of other PKC*δ* splice variants are not yet established. Our previous studies in neurons have demonstrated that the prosurvival variant of PKC*δ* increases the expression of Bcl2 and Bcl-xL which are known to increase cellular survival [16].

Obesity is associated with a chronic low inflammatory state due to the changes in secretory functions of the adipocytes. Enlarged adipocytes (hypertrophy) lead to increased adipocyte-derived free fatty acids which stimulate macrophages. In severely obese patients, macrophages invade the adipose tissue and release chemokines leading to necrosis and autophagy of adipocytes [17]. These complications add to obese-associated metabolic syndrome, diabetes, insulin resistance, and hepatic steatosis. These forms of cell death are distinct from apoptosis (programmed cell death) in adipocytes. Apoptosis is shown to be substantially greater in omental depots compared to subcutaneous depots in healthy individuals [18]. Here, we show that obese samples are resistant to apoptosis and express the antiapoptosis genes fourfold greater than in lean samples. This may explain the increased difficulty to lose weight and maintain the weight loss in overweight and obese individuals. Larger adipocytes are an indicator of type II diabetes independent of insulin resistance [19]. Previous studies showed that PPAR*γ* and adiponectin expression was lower in obesity and type II diabetes [20, 21]. Our data indicated that obese patients have dysregulated adipogenesis program. This may be modulated by the signaling kinase PKC*δ*VIII which is overexpressed in obese individuals. Our data also indicated dysregulated alternative splicing of apoptosis genes in obese differentiating cells. This aberrant molecular profile may be a causative effect leading to obesity or may be a consequence of obesity and its comorbidities. We are evaluating this further in our laboratory. Here, we have defined the differences in gene expression between lean and obese adipocytes.

In conclusion, it may be possible to modulate alternative splicing during adipogenesis for therapeutic treatments of obesity. Our results suggest a novel role for a key signaling kinase PKC*δ*VIII in the process of adipogenesis. Expression levels of PKC*δ*VIII splice variant are increased in obesity and may be linked to the cause or consequence of dysregulated adipogenesis in obese patients. Since new adipocytes are required for the maintenance and the endocrine function of adipocytes, balancing the gene expression of apoptotic genes may be a better approach rather than complete inhibition of adipogenesis.

## References

[1] Rodríguez-Acebes S, Palacios N, Botella-Carretero JI (2010). Gene expression profiling of subcutaneous adipose tissue in morbid obesity using a focused microarray: distinct expression of cell-cycle- and differentiation-related genes. *BMC Medical Genomics*.

[2] Guzey M, Jukic D, Arlotti J, Acquafondata M, Dhir R, Getzenberg RH (2004). Increased apoptosis of periprostatic adipose tissue in VDR null mice. *Journal of Cellular Biochemistry*.

[3] Fischer-Posovszky P, Tornqvist H, Debatin KM, Wabitsch M (2004). Inhibition of death-receptor mediated apoptosis in human adipocytes by the insulin-like growth factor I (IGF-I)/IGF-I receptor autocrine circuit. *Endocrinology*.

[4] Ishiko O, Sumi T, Yoshida H, Hyun Y, Ogita S (2001). Comparison of expression of apoptosis regulatory proteins in the adipose tissue of tumor-bearing and diet-restricted rabbits. *International Journal of Molecular Medicine*.

[5] Della-Fera MA, Choi YH, Hartzell DL, Duan J, Hamrick M, Baile CA (2005). Sensitivity of ob/ob mice to Leptin-induced adipose tissue apoptosis. *Obesity Research*.

[6] Kim DH, Woods SC, Seeley RJ (2010). Peptide designed to elicit apoptosis in adipose tissue endothelium reduces food intake and body weight. *Diabetes*.

[7] Earnshaw WC, Martins LM, Kaufmann SH (1999). Mammalian caspases: structure, activation, substrates, and functions during apoptosis. *Annual Review of Biochemistry*.

[8] Jiang K, Apostolatos AH, Ghansah T (2008). Identification of a novel antiapoptotic human protein kinase C *δ* isoform, PKC*δ*VIII in NT2 cells. *Biochemistry*.

[9] Apostolatos H, Apostolatos A, Vickers T (2010). Vitamin A metabolite, all-trans-retinoic acid, mediates alternative splicing of protein kinase C *δ*VIII (PKC*δ*VIII) isoform via splicing factor SC35. *Journal of Biological Chemistry*.

[10] Jun DY, Han CR, Choi MS, Bae MA, Woo MH, Kim YH (2011). Effect of mollugin on apoptosis and adipogenesis of 3T3-L1 preadipocytes. *Phytotherapy Research*.

[11] Kim SM, Park HS, Jun DY (2009). Mollugin induces apoptosis in human Jurkat T cells through endoplasmic reticulum stress-mediated activation of JNK and caspase-12 and subsequent activation of mitochondria-dependent caspase cascade regulated by Bcl-xL. *Toxicology and Applied Pharmacology*.

[12] Patel NA, Song SS, Cooper DR (2005). PKC*δ* alternatively spliced isoforms modulate cellular apoptosis in retinoic acid-induced differentiation of human NT2 cells and mouse embryonic stem cells. *Gene Expression*.

[13] Peluso JJ, Pappalardo A, Fernandez G (2001). Basic fibroblast growth factor maintains calcium homeostasis and granulosa cell viability by stimulating calcium efflux via a PKC*δ*-dependent pathway. *Endocrinology*.

[14] Kilpatrick LE, Lee JY, Haines KM, Campbell DE, Sullivan KE, Korchak HM (2002). A role for PKC-*δ* and PI 3-kinase in TNF-*α*-mediated antiapoptotic signaling in the human neutrophil. *The American Journal of Physiology*.

[15] Zrachia A, Dobroslav M, Blass M (2002). Infection of glioma cells with Sindbis virus induces selective activation and tyrosine phosphorylation of protein kinase C *δ*: implications for sindbis virus-induced apoptosis. *Journal of Biological Chemistry*.

[16] Apostolatos A, Song S, Acosta S (2012). Insulin promotes neuronal survival via the alternatively spliced protein kinase CdeltaII isoform. *Journal of Biological Chemistry*.

[17] Strissel KJ, Stancheva Z, Miyoshi H (2007). Adipocyte death, adipose tissue remodeling, and obesity complications. *Diabetes*.

[18] Papineau D, Gagnon A, Sorisky A (2003). Apoptosis of human abdominal preadipocytes before and after differentiation into adipocytes in culture. *Metabolism*.

[19] Weyer C, Foley JE, Bogardus C, Tataranni PA, Pratley RE (2000). Enlarged subcutaneous abdominal adipocyte size, but not obesity itself, predicts type II diabetes independent of insulin resistance. *Diabetologia*.

[20] Tankó LB, Siddiq A, Lecoeur C (2005). ACDC/adiponectin and PPAR-*γ* gene polymorphisms: implications for features of obesity. *Obesity Research*.

[21] Ouchi N, Kihara S, Funahashi T, Matsuzawa Y, Walsh K (2003). Obesity, adiponectin and vascular inflammatory disease. *Current Opinion in Lipidology*.

